# A Fiber-Optic Interferometric Tri-Component Geophone for Ocean Floor Seismic Monitoring

**DOI:** 10.3390/s17010047

**Published:** 2016-12-28

**Authors:** Jiandong Chen, Tianying Chang, Qunjian Fu, Jinpeng Lang, Wenzhi Gao, Zhongmin Wang, Miao Yu, Yanbo Zhang, Hong-Liang Cui

**Affiliations:** 1 College of Instrumentation & Electrical Engineering, Jilin University, Changchun 130061, China; chenjd13@mails.jlu.edu.cn (J.C.); fuqj16@mails.jlu.edu.cn (Q.F.); langjp14@mails.jlu.edu.cn (J.L.); gaowz15@mails.jlu.edu.cn (W.G.); miaoyu14@mails.jlu.edu.cn (M.Y.); hcui@jlu.edu.cn (H.-L.C.); 2 Institute of Automation, Shandong Academy of Sciences, Jinan 250014, China; gezhb@sdas.org (Z.W.); zhangyb@sdas.org (Y.Z.)

**Keywords:** fiber optic Michelson interferometer, vibration sensor, phase generated carrier, geophone

## Abstract

For the implementation of an all fiber observation network for submarine seismic monitoring, a tri-component geophone based on Michelson interferometry is proposed and tested. A compliant cylinder-based sensor head is analyzed with finite element method and tested. The operation frequency ranges from 2 Hz to 150 Hz for acceleration detection, employing a phase generated carrier demodulation scheme, with a responsivity above 50 dB re rad/g for the whole frequency range. The transverse suppression ratio is about 30 dB. The system noise at low frequency originated mainly from the 1/f fluctuation, with an average system noise level −123.55 $\mathrm{dB}\;\mathrm{re}\;\mathrm{rad}/\sqrt{\mathrm{Hz}}$ ranging from 0 Hz to 500 Hz. The minimum detectable acceleration is about 2 $\mathrm{ng}/\sqrt{\mathrm{Hz}}$, and the dynamic range is above 116 dB.

## 1. Introduction

Submarine seismic and tsunami observation networks recently implemented around the world have strengthened the capability in monitoring, early warning, and forecasting of such natural disasters. These installations include NEPTUNE, VENUS, Canada’s observation networks [1,2]; submarine seismic and tsunami observation networks of New Zealand [3] and Japan [4]; and the advanced national seismic system (ANSS) of the United States. Geophones, water level gauges, and hydrophones are the core sensory components of these observation networks [5,6,7,8]. For submarine seismic monitoring, it is more valuable to detect long-distance ground vibration waves, especially the low frequency seismic waves. The fiber-optic geophone technology is originally developed for the US Navy [9,10,11], and it has proved to be much more reliable and sensitive than the older systems which were piezo-electric-based electronic/digital sensors. Consequently, these sensors are at the focal points of major research efforts around the world. Precise and reliable detection of seismic waves is the key to any seismic observation network. Fiber-optic-based seismometers have been commonly used in existing observation networks. As with any fiber optic sensors, they are immune to electromagnetic interference [12], with high sensitivity, low noise floor, stable and predictable temperature performance, and a host of other positive attributes, they are ideal for networking, long distance, and long term monitoring applications.

In this paper, we focus on the application of fiber optic interferometric vibration detection schemes potentially applicable for submarine seismic monitoring. In 1987, Gardner et al. first reported a mandrel structure fiber-optic interferometer seismometer, which had a detection threshold of 1 $\mathrm{ng}/\sqrt{\mathrm{Hz}}$ within the detection frequency band of 10–1000 Hz [11]; in 1990, Brown et al. reported a plate structure seismometer whose application frequency was above 20 Hz [13]; in 2002, Shindo et al. reported a seismometer with a plate structured fiber-optic interferometer, which was applied for vibration frequency above 3 Hz; in 2007, Beverini et al. reported a low-frequency sensing system whose low frequency limit was 0.05 Hz [14]; in 2011, Lin et al. realized a low frequency vibration detection scheme which had the lowest frequency of 0.01 Hz produced by piezoelectric optic fiber stretcher, after comparing two demodulation schemes for phase generated carrier (PGC) [15]. Zeng et al., reported a three-component fiber-optic accelerometer for well logging in 2004 [16] with the frequency range 3–800 Hz, applied to well logging without considering the optical polarization fade in the interferometer. Moreover, Jia et al. reported a self-calibrated non-contact fiber-optic Fabry-Perot interferometric vibration displacement sensor, which had a nonlinearity 0.29% and less than 2.06 nm resolution with a feedback interferometric arm length control [17], although it was not suitable for high-amplitude and low-frequency vibration monitoring; while others reported various schemes for demodulation of the vibration signals [18,19,20,21,22]. In order to obtain accurate measurements of external vibrations, a feedback control element, such as a piezoelectric fiber stretcher, is usually embedded in one of the interferometric arms. However, such an implement limits the sensing length and hampers the development and application of all fiber sensing networks based on such sensory nodes.

In the present paper, we report on a tri-component geophone based on Michelson interferometry, with a compliant cylinder-based sensor head design. Theoretical and experimental results show that the new optic fiber geophone can fulfill the demand of applications in all fiber observation networks for submarine seismic monitoring, in terms of an adequate operation frequency range, high responsivity, high transverse suppression ratio, low system noise floor, and a system design without the need of a feedback control element in the interference arms.

The structure of this paper is as follows. The demodulation principle for phase generated carrier and finite element analysis for the compliant cylinder vibration accelerometer are presented in Section 2. The experimental results and discussion are shown in Section 3, and the conclusion is given in Section 4.

## 2. Demodulation Algorithm and Analysis for Accelerometer

**Figure 1:**
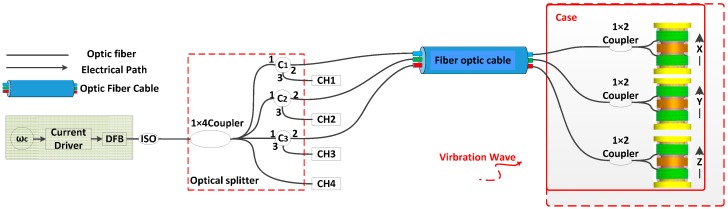
Tri-component optic fiber interference vibration detection system. $\omega_{c}$: Cosine signal with frequency of $\omega_{c}$; DFB: distribute feedback laser; ISO: fiber-optic isolator; C1–C4: Circulator1–Circulartor4; CH1–CH4: four channels’ photodetectors and processor modules.

Our design is based on an optical fiber Michelson interferometer to realize vibration detection, and align three nominally identical sensors in the three orthogonal directions to detect the vibration signals simultaneously (see Figure 1). Performance of a direct-modulation distributed feedback (DFB) semiconductor laser is dependent on the driving current. The output of the laser, whose power is modulated by a sinusoidal current with frequency $\omega_{c}$, is coupled into four light paths; the first three paths are transferred into the sensor head via an optic fiber cable; the fourth path is connected with a photodiode. There is a 1 × 2 optical fiber coupler in each path in the sensor, where the light is split into two beams via the coupler, and reflected by the Faraday mirror which can eliminate the polarization fade of the interferometer at the end. Optical interference takes place in the coupler and is transferred back to the photodetector through the optic fiber cable and the circulator (one in each of the three sensing channels). Optic fibers from the couplers in the sensing channels are wound around the compliant cylinders, which are made of silicon rubber, constituting the interference arms. Vibration disturbances will set the inertial mass into relative motion up and down, which produces a push-pull on the pair of fiber winding above and below the inertia mass block. This in turn will act on the compliant cylinder and change the lengths of the optic fibers, inducing a time variation in the interference paths. The vibration information can be demodulated from the optic interference signal by a modulation model in each channel. Three demodulation channels monitor the three orthogonally oriented sensors and form a tri-component sensor head.

### 2.1. Demodulation of Direct Modulation Phase Generated Carrier

**Figure 2:**
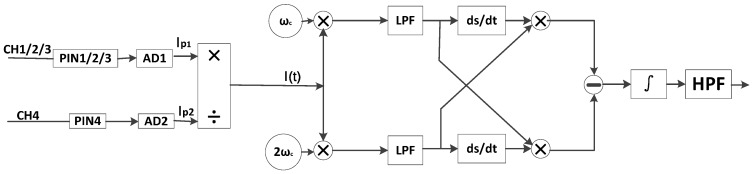
Differential cross multiplication demodulation. PIN1/2/34: photodetector of channel 1/2/3/4; AD1/2: analog to digital convert module; I(t): the interferometric signal; $\omega_{c}$: Cosine signal with frequency of $\omega_{c}$; $2\omega_{c}$: Cosine signal with frequency of $2\omega_{c}$; LPF: low pass filter; ds/dt: differential operation; *∫*: integral operation; HPF: high pass filter.

There are two generally adopted methods for demodulation of phase generated carrier (PGC) modulation: differential cross multiplication (DCM) demodulation, and arctangent demodulation [15,22,23], along with several improvements suggested over the years [17,24]. In this paper, we consider the direct modulation phase generated carrier (D-PGC) demodulation by the DCM scheme in each sensing channel, as illustrated in Figure 2.

The optical power output of the DFB laser, modulated by a sinusoidal driving current with amplitude $i_{c}$ and modulation frequency $\omega_{c}$, is given by

(1) $$I_{0}(t)=I_{0}+I_{0}m\cos\omega_{c}t,$$

where $I_{0}$ is the un-modulated light power of the DFB laser, and m is the modulation amplitude coefficient of the optical power.

Optical interference is formed in the coupler within the sensor case and expressed as

(2) $$I_{d}(t)=\varsigma I_{0}(t)+I_{0}(t)\varsigma k\cos(\varphi_{c}(t)+\varphi_{s}(t)+\varphi_{0}),$$

where ς is the attenuation coefficient of the light path, *k* (*k <* 1) is the fringe visibility of the optical interference which is mainly affected by the optical polarization, $\varphi_{c}(t)$ is the optical phase change introduced by the modulation, $\varphi_{s}(t)$ is the optical phase change caused by ambient vibration under detection, $\varphi_{0}$ is the initial optical phase difference mainly caused by unbalance in the interference arms, and is generally considered to be constant. $\varphi_{s}(t)$ and $\varphi_{c}(t)$ are given by

(3) $$\varphi_{s}(t)=A_{s}\cos(\omega_{s}t+\delta ),$$

(4) $$\varphi_{c}(t)=C\cos(\omega_{c}t),$$

where *A_s_* is the amplitude of the optical phase change produced by the ambient vibration, $\omega_{s}$ is the angular frequency of the optical phase change by the ambient vibration, and δ is the initial phase of it. *C* is the amplitude of the optical phase change, and $\omega_{c}$ is the angular frequency of the optical phase change due to the modulation.

The signal from the photodetector in channel 1 is the optical interference signal multiplied by an optical-electrical conversion coefficient α (α<1): and the signal from the photodetector in channel 4 is the laser output light power multiplied by an optical-electrical conversion coefficient β, i.e.,

(5) $$I_{P1}(t)=\alpha I_{d}(t),$$

(6) $$I_{P2}(t)=\beta I_{0}(t),$$

According to Equations (2), (5) and (6), the effect of the optical power modulation on the interference could be eliminated after dividing Equation (5) by Equation (6), and the optical interference signal can be re-expressed as

(7) $$I(t)=\gamma +\gamma k\cos(\varphi_{c}(t)+\varphi_{s}(t)+\varphi_{0}),$$

where γ=αd/β is a constant after establishing the optical path and the electrical path. Comparing Equation (7) with Equation (2), the power modulation of laser output light is eliminated in theory. Separating the second term from Equation (7) and combining with the first kind of Bessel function series, $\cos\varphi_{c}(t)$ and $\sin\varphi_{c}(t)$ can be expressed as Equations (8) and (9).

(8) $$\cos(\varphi_{c}(t))=\cos(C\cos\omega_{c}t)=\left\{J_{0}(C)+2\sum_{m=1}^{+\infty }{\left(-1\right)}^{m}J_{2m}(C)\cos2m\omega_{c}t\right\},$$

(9) $$\sin(\varphi_{c}(t))=\sin(C\cos\omega_{c}t)=\left\{-2\sum_{m=1}^{+\infty }{\left(-1\right)}^{m}J_{2m-1}(C)\cos(2m-1)\omega_{c}t\right\}.$$

**Figure 3:**
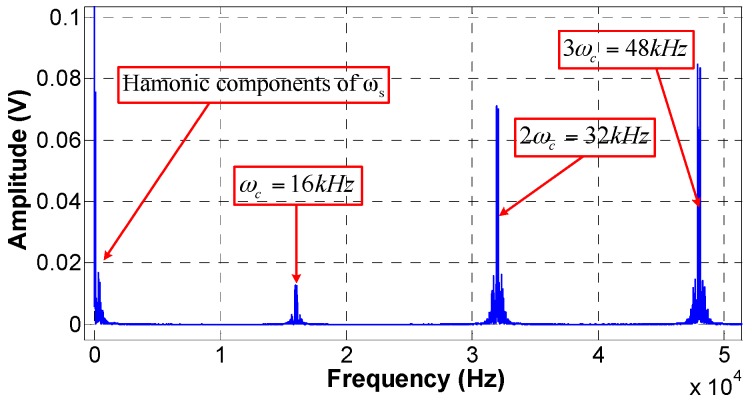
Photodetector signal of optical interference within channel 1 in the frequency domain.

Equation (8) is consisted of constant component and even-order harmonics of $\omega_{c}$. Equation (9) is made up of odd-order harmonics of $\omega_{c}$. $\cos(\varphi_{s}(t)+\varphi_{0})$ and $\sin(\varphi_{c}(t)+\varphi_{0})$ can be expanded similarly. Thus, the optical interference signal is consisted of harmonic frequencies of $\omega_{c}$ and harmonic frequencies of $\omega_{s}$, as well as sum frequency and difference frequency of them. The photodetector signal of the optical interference in frequency domain is presented in Figure 3.

**Figure 4:**
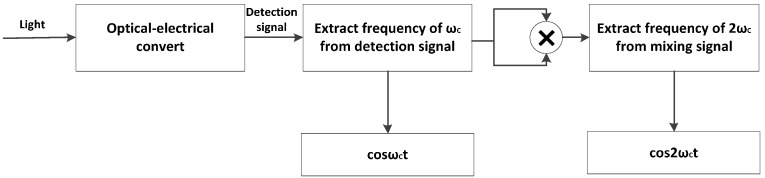
The progress of extracting the signal in frequency domain for mixing.

Demodulating the signal $\varphi_{s}(t)$ starts with mixing the interference signal with two separated sinusoidal reference frequencies of $\omega_{c}$ and $2\omega_{c}$, followed by extracting the sinusoidal signal with frequency of $\omega_{c}$ from the photodetector signal in channel 4, and the sinusoidal signal with a frequency of $2\omega_{c}$ from another mixing signal $\omega_{c}$ multiplying itself, this progress uses a fast Fourier transform (FFT) filter to multiply the photodetector signal in frequency domain, which does not induce signal delays in time domain and is known to entail highly efficient performance. The scheme is shown in Figure 4.

After a low-pass filter, whose low pass cutoff frequency is set to half of the modulation frequency ($\omega_{c}/2$), the result of the differential cross multiplication (DCM) and high pass filter which isolate the DC component can be expressed as

(10) $$S_{4}=B\cos(\omega_{s}t+\delta ),$$

where the coefficient $B=A_{s}J_{1}(C)J_{2}(C)$. Comparing the result of demodulation (Equation (10)) and the variable phase (Equation (3)) which is to be detected, the only difference lies in the amplitudes, therefore signal demodulation has been achieved.

### 2.2. Design of Detector

**Figure 5:**
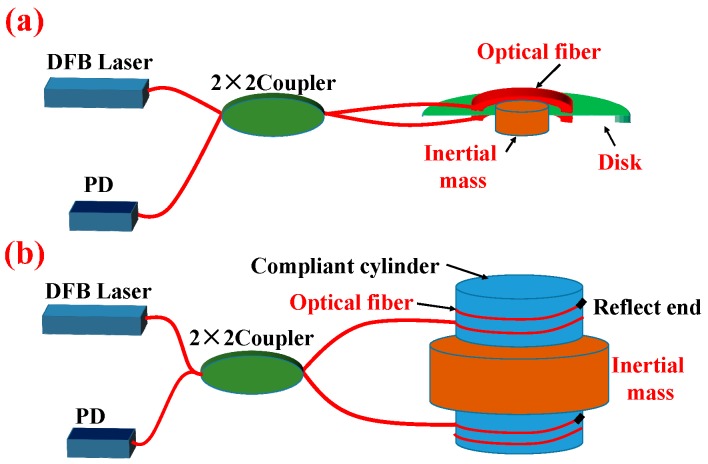
Two types of optical fiber interference vibration detector based on Michelson interferometer. (**a**) Disk type Michelson interferometer; (**b**) Compliant cylinder type Michelson interferometer.

There are two commonly adopted structures for sensor head design based on Michelson interferometer, disk type and compliant cylinder type [25,26]. The sketch of the two apparatuses is shown in Figure 5.

The disk type optical fiber interference detector mainly consists of a soft disk, an inertial mass, and two optical fibers, which are circularly glued on either surfaces of the disk, which is usually made of high elasticity manganese steel. The compliant cylinder type is mainly made up of an inertial mass, and two compliant cylinders. The ends of the sensing fibers of both types of detectors are terminated with an anti-reflective mirror or a Faraday mirror.

For both types of sensor heads configured as a Michelson interferometer, the mechanical models of the detectors are basically a damped mass-spring oscillator. When disturbed, the inertial mass moves with the vibration wave, which pulls and pushes the disk or compliant cylinder, causing the lengths of the optical interferometer arms to change; the photodetector (PD) converts the light signals into electrical current signals to be demodulated. The disk type optical fiber interference detector has a structural weakness, in that the optical fiber circularly glued on the surface is subject to deterioration of the glue, which could affect the performance of the disk based sensor design. In addition, in order to achieve high sensitivity, the disk area must be large enough to accommodate long optical fiber winding. Moreover, the Faraday rotated mirror at the interferometer end can eliminate the visibility of the interferometer caused by optic polarization differences [27].

**Figure 6:**
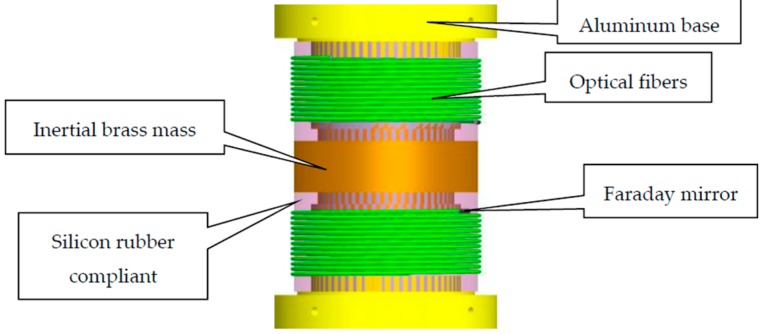
The structure of the compliant cylinder geophone.

The design of the compliant cylinder is shown in Figure 6, where the compliant cylinder is made of silicon rubber (Type-601 silicon rubber, Beijing Hagibis Technology Co., Ltd., Beijing, China); the inertial mass made of brass; the sensing optical fiber is Corning G657.A2 single-mode communication fiber (Corning, New York, NY, USA). The physical characteristics of the materials are listed in Table 1. The optical fiber is wound around the compliant cylinder. To ensure good coupling between the optical fiber and the compliant cylinder, the fiber is sealed with a thin layer of silicon rubber again.

**Figure 7:**
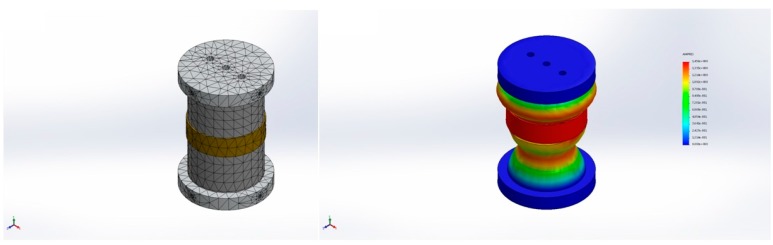
The mesh and the result for FEM simulation.

Numerical simulation of the natural frequency for the compliant cylinder used in sensing vibration had been carried out using the finite element method (FEM). The simulation parameters were based on those listed in Table 1; the mesh used and the result of the simulation are shown in Figure 7.

The Young’s modulus of the combination compliant cylinder is taken to be 1.55 × 10^6^ N/m^2^, resulting in a theoretical natural frequency of 258.38 Hz.

## 3. Experimental Results and Discussion

**Figure 8:**
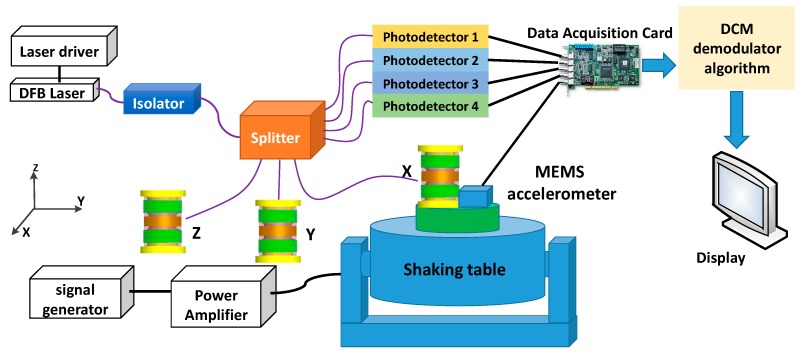
Equipment of experiment system for vibration measurement.

The testing fixture for the performance of the geophone is shown in Figure 8. It consists of an all fiber geophone system with three geophones, a shaking table, and a comparable MEMS accelerometer (SILICON DESIGNS, Model 2220, Silicon Designs, Inc., Kirkland, WA, USA). The experiment tested five parameters of the geophone: the operation frequency, the responsivity, the transverse suppression ratio, the minimum detectable signal level, and the dynamic range.

The vibration platform can provide the excitation force, whose maximum is more than 50 N, and the output signal of it ranges from 2 to 5 kHz, which is suitable for the low frequency vibrating detection. The DFB laser is direct-modulated; the light from the laser is separated by the optical splitter and coupled into the four optical paths, and the first three paths are transferred into detectors. The fourth path is connected with a photodiode, which monitors the laser output light power; the optical interference signals carrying vibration information are detected by photodiodes 1–3. After the demodulation module, which is programmed in LABVIEW and MATLAB, the vibration signals are displayed by a computer.

### 3.1. Responsivity and Operation Range of Frequency

**Figure 9:**
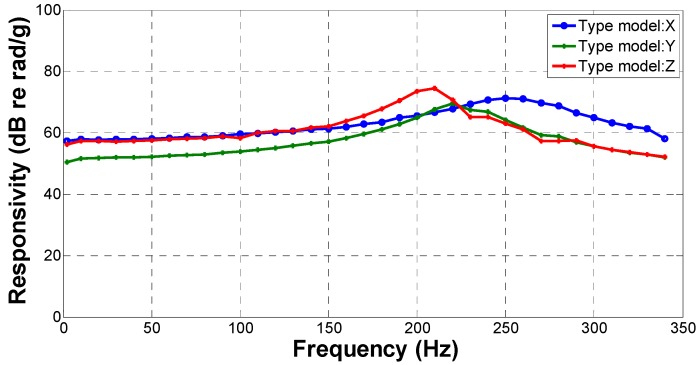
Responsibility test results for the three orthogonal geophones.

The sensitivity of the geophone is defined as the ratio of the demodulation optical phase shift ∆φ (rad) to the input acceleration amplitude a (g), and it is usually related to the responsivity in the photodetector. The sensor with the compliant cylinder structure responds linearly in frequency below the sensor’s resonance [11,28]. Thus the sensitivity of the sensor is expressed as

(11) $$R_{e}=\frac{\Delta \varphi }{a},$$

where ∆φ is the phase shift induced by the acceleration a which is detected by a standard commercial MEMS accelerometer in our measurement, with the test results shown in Figure 9.

The result in Figure 9 shows that the minimum test responsivity on the three orthotropic directions is 57.4 dB re rad/g, 50.45 dB re rad/g, and 56.21 dB re rad/g in the x, y, and z direction, respectively. The corresponding resonance frequency of geophones is 250, 220 and 210 Hz. They are close to the theoretical value of 258.38 Hz, with the difference mainly caused by the coupling ratio between the compliant cylinder and the optic fiber. The geophones act as accelerators when they are operated below the resonance frequency, and the test result shows that the tri-component geophone has a responsivity higher than 53 dB re rad/g when operated in the frequency range from 2 Hz to 200 Hz. The mean value of the responsivity below 150 Hz is 59.04, 53.44 and 58.74 dB re rad/g, in the three orthogonal directions, respectively. The responsivity value is in the range of 2–150 Hz less fluctuation which are 3.13, 3.66 and 2.64 dB re rad/g, respectively, thus the operated frequency range of the geophone is from 2 Hz to 150 Hz.

### 3.2. Transverse Suppression Ratio

The transverse suppression ratio is defined by the ratio of the demodulation phase results when the geophone is mounted on the shaking table in two orthotropic directions, while the amplitude and frequency of shaking are kept the same. The averages of the transverse suppression for the geophones are 32.64, 29.76, and 28.57 dB, ranging from 2 Hz to 150 Hz. The typical low frequency experimental results for acceleration value are independently obtained by the MEMS accelerometer, which is 0.217 g at 10 Hz. The measured results for the transverse suppression ratio of the optic fiber interferometric geophones are listed in Table 2.

From the test results in Table 2, we conclude that the tri-component geophones have transverse suppression ratios of about 30 dB, ensuring that the geophones have good directional property. This latter property is important in discriminating between longitudinal and transverse seismic waves, and consequently in locating the vibration source.

### 3.3. Minimum Detectable Signal Level and Dynamic Range

The minimum detectable acceleration level of the optic fiber geophone system is evaluated by the noise floor of the system and expressed as

(12) $$a_{\min}=\frac{System\;noise\;floor(\mathrm{rad}/\sqrt{\mathrm{Hz}})}{Geophone\;phase\;responsivity(\mathrm{rad}/g)}.$$

**Figure 10:**
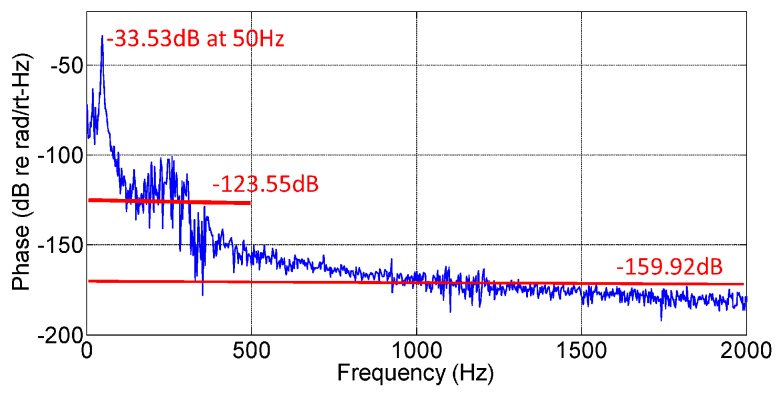
Noise of the geophone system output.

The detected noise signal in frequency domain of the geophone is shown in Figure 10.

The average system noise level of the geophone system is measured in frequency domain, and defined with the mean power density of the geophone signal in the detectable frequency band but without an input signal. The sample frequency of the data acquisition card is set to $1\times 10^{6}\;Sa/s$, setting the actual upper frequency at $5\times 10^{5}\;\mathrm{Hz}$ by Nyquist’s Theorem. However, only with the shaking signal frequency in the region of 0–150 Hz does the sensor operate as an accelerometer, with its acceleration output linearly dependent on the input shaking signal. Far below the Nyquist frequency. The experimental results in Figure 10 show that the system noise complies with the 1/f fluctuation at the low frequency region, which means the geophone system has a higher noise level in the low frequency range, having values below −150 $\mathrm{dB}\;\mathrm{re}\;\mathrm{rad}/\sqrt{\mathrm{Hz}}$ (at 500 Hz). Taken together, the calculation for the average system noise level has two experimental results, −123.55 $\mathrm{dB}\;\mathrm{re}\;\mathrm{rad}/\sqrt{\mathrm{Hz}}$ from 0 Hz to 500 Hz, and −159.92 $\mathrm{dB}\;\mathrm{re}\;\mathrm{rad}/\sqrt{\mathrm{Hz}}$ from 0 Hz to 2000 Hz, respectively. The minimum detectable accelerations in the frequency range from 0 Hz to 500 Hz for the tri-component geophone can be calculated by using Equation (12) and are listed in Table 3.

The results in Table 3 show that the geophone system has the minimum detectable acceleration level $a_{min}\approx 2\;\mathrm{ng}/\sqrt{\mathrm{Hz}}$, while the average maximum variable phase amplitude of the geophones is 747.7 rad with 1 g acceleration amplitude with frequency ranging from 2 Hz to 150 Hz; the dynamic range of the geophones are 119.25 dB, 116.58 dB, and 118.58 dB, respectively for the three components.

As noted above, differences in responsivity of the orthogonal components of the geophones were observed in the experiments, which were mainly caused by variations of the coupling ratio between the compliant cylinder and the optic fiber. The responsivity difference between the X model and Y model geophone is decreased from 6.95 dB to 4.28 dB as the shaking frequency increases from 2 Hz to 150 Hz; the responsivity difference between the Y model and Z model geophone is decreased from 6.95 dB to 4.28 dB; the responsivity difference between the X model and Z model geophone is decreased from 1.19 dB to 0 dB. It is apparent that the low frequency consistency between the geophones needs to be improved. Employment of an electromechanical fiber optic winding machine may alleviate this problem to some extent. The low frequency characteristics of the geophones at vibration frequencies below 1 Hz cannot be quantified at this time, due to the restrictions of testing fixtures and signal process. On the other hand, when the tri-component fiber optic geophones are well encapsulated and mounted on the ocean floor, where the environment temperature is relatively stable, the effect of the temperature variation (which had some minor effect in our laboratory tests) should be tolerable for the push and pull structural design of the geophone.

## 4. Conclusions

In summary, the principle of the optical fiber Michelson-interference-compliant cylinder geophone was described and experimentally demonstrated. A direct modulation scheme was introduced to realize the structure of an all optical fiber system for vibration detection. The scheme of differential cross multiplication (DCM) was analyzed in detail, the mixing signals were extracted in the frequency domain which does not induce signal delay in the time domain in general and features highly efficient performance with the tri-component geophone. The compliant cylinder geophone is analyzed using the finite element method, predicting a resonance frequency 258.38 Hz, which is tested by the experimental results. The operation frequency ranges from 2 Hz to 150 Hz for acceleration detection, and the responsivity of the geophones are all above 50 dB re rad/g. The transverse suppression ratios are about 30 dB. The system noise complies with the 1/f fluctuation and the average of the system noise level is −123.55 $\mathrm{dB}\;\mathrm{re}\;\mathrm{rad}/\sqrt{\mathrm{Hz}}$, in the frequency band from 0 Hz to 500 Hz; the minimum detectable acceleration is about 2 $\mathrm{ng}/\sqrt{\mathrm{Hz}}$, and the dynamic range is above 116 dB.

Such a tri-component fiber optic geophone is potentially suitable for ocean floor seismic event monitoring. The next research focus of ours will be on improving the uniformity of performance of all three components, partially by perhaps employing an electromechanical fiber optic winding machine and improving the algorithms and signal processing, especially in reducing sample frequency for lower frequency vibration detection.

## Figures and Tables

**Table 1:** Physical characteristics of the sensor head materials.

Parts	Symbols	Parameters	Value (Units)
Inertial brass mass	ρ_1_	Density	8500 (kg/m^3^)
	m_1_	Mass	412.5 (g)
	E_1_	Young’s modulus	1 × 10^11^ (N/m^2^)
	δ_1_	Poisson ratio	0.33
Optical fibers	D_2_	Diameter	242 (um)
	L_2_	Length of fiber	10 (m)
	E_2_	Young’s modulus	7.3 × 10^10^ (N/m^2^)
	δ_2_	Poisson ratio	0.17
Compliant cylinder	m_3_	Mass	59.0 (g)
	ρ_3_	Density	1246.5 (kg/m^3^)
	E_3_	Young’s modulus	1.55 × 10^6^ (N/m^2^)
	δ_3_	Poisson ratio	0.48
	δ_b_	Tensile strength	6 × 10^6^ (N/m^2^)
Aluminum base	ρ_4_	Density	2700 (kg/m^3^)
	m_4_	Mass	110.1 (g)
	E_4_	Young’s modulus	6.9 × 10^10^ (N/m^2^)
	δ_4_	Poisson ratio	0.33

**Table 2:** Transverse suppression ratio test results for the geophone.

Model	The Average of the Transverse Suppression (2–150 Hz)	Transverse Suppression Ratio at 10 Hz
X	32.64 dB	34.61 dB
Y	29.76 dB	32.20 dB
Z	28.57 dB	30.77 dB

**Table 3:** The minimum detectable acceleration of the tri-component geophone.

Model	The Minimum Detectable Acceleration
X	$0.90\times 10^{-9}g/\sqrt{\mathrm{Hz}}$
Y	$2.00\times 10^{-9}g/\sqrt{\mathrm{Hz}}$
Z	$1.03\times 10^{-9}g/\sqrt{\mathrm{Hz}}$

## References

[1] Rogers G.C., Meldrum R., Mulder T.R., Baldwin R., Rosenberger A., Moran S., Beeler N.M. (2010). First observations form the NEPTUNE Canada seismograph network. Seismol. Res. Lett..

[2] Moran K. (2013). Canada’s cabled ocean networks humming along. Eos Trans. Am. Geophys. Union.

[3] Cavill A.W., Cassidy J., Brennan B.J. (1997). Results from the new seismic monitoring network at Egmont Volcano, New Zealand: Tectonic and hazard implications. N. Z. J. Geol. Geophys..

[4] Okada Y., Kasahara K., Hori S., Obara K., Sekiguchi S., Fujiwara H., Yamamoto A. (2004). Recent progress of seismic observation network in Japan. Earth Planets Space.

[5] Cranch G.A., Nash P.J., Kirkendall C.K. (2003). Large-Scale Remotely Interrogated Arrays of Fiber-optic Interferometric Sensors for Underwater Acoustic Applications. IEEE Sens. J..

[6] Gulaev A.Y., Kitaev S.M., Listvin V.N., Potapov V.T., Sedykh D.A., Shatalin S.V., Yushkaĭtis R.V. (1990). Deep-water fiber-optic hydrophone. Sov. J. Quantum Electron..

[7] Kirkendall C.K., Dandridge A. (2004). Overview of high performance fiber-optic sensing. J. Phys. D Appl. Phys..

[8] Shindo Y., Yoshikawa T., Mikada H. A large scale seismic sensing array on the seafloor with fiber optic accelerometers. Proceedings of the IEEE Sensors 2002.

[9] Kersey A.D., Jackson D.A., Corke M. (1982). High-sensitivity fiber-optic accelerometer. Electron. Lett..

[10] Mills G.B., Garrett S.L., Carome E.F. (1984). Fiber-optic gradient hydrophone. Tech. Symp. East.

[11] Gardner D.L., Hofler T., Baker S.R., Yarber R., Garrett S. (1987). A fiber-optic interferometer seismometer. J. Light Technol..

[12] Björn N.P.P., Toko J.L., Thornburg J.A., Slopko F., He R., Zhang C.H. A high performance fiber optic seismic sensor system. Proceedings of the Thirty-Eighth Workshop on Geothermal Reservoir Engineering Stanford University.

[13] Brown D.A., Garrett S.L. (1990). An interferometric fiber optic accelerometer. SPIE.

[14] Beverini N., Maccioni E., Morganti M., Stefani F., Falciai R., Trono C. (2007). Fiber laser strain sensor device. J. Opt. A Pure Appl. Opt..

[15] Wang L., He J., Lin F., Liu Y. (2011). Ultra low frequency phase generated carrier demodulation technique for fiber sensors. Chin. J. Lasers.

[16] Zeng N., Shi C.Z., Zhang M., Wang L.W., Liao Y.B., Lai S.R. (2004). A 3-component fiber-optic accelerometer for well logging. Opt. Commun..

[17] Jia P.G., Wang D.H. (2012). Self-calibrated non-contact fibre-optic Fabry-Perot interferometric vibration displacement sensor system using laser emission frequency modulated phase generated carrier demodulation scheme. Meas. Sci. Technol..

[18] Shi Q., Tian Q. (2010). Performance improvement of phase-generated carrier method by eliminating laser-intensity modulation for optical seismometer. Opt. Eng..

[19] Tong Y., Zeng H., Li L., Zhou Y. (2012). Improved phase generated carrier demodulation algorithm for eliminating light intensity disturbance and phase modulation amplitude variation. Appl. Opt..

[20] Chen Y., Wang J., Luo H., Meng Z. (2014). Research of the polarity characteristic for an optical fiber accelerometer demodulated by phase generated carrier technology. Optik.

[21] He J., Wang L., Li F., Liu Y. (2010). An ameliorated phase generated carrier demodulation algorithm with low harmonic distortion and high stability. J. Lightwave Technol..

[22] Huang S.-C., Huang Y.-F., Hwang F.-H. (2013). An improved sensitivity normalization technique of PGC demodulation with low minimum phase detection sensitivity using laser modulation to generate carrier signal. Sens. Actuators A.

[23] Christian T.R., Frank P.A., Houston B.H. (1994). Real-time analog and digital demodulator for interferometer fiber optic sensors. Proc. SPIE.

[24] Wang Z., Luo H., Xiong S., Ni M., Hu Y. (2007). A J0-J1 method for measurement of dunamic phase changes in an interferometric fiber sensor. Chin. J. Lasers.

[25] Shajenko P., Flatley J.P., Moffett M.B. (1978). On fiber-optic hydrophone sensitivity. J. Acoust. Soc. Am..

[26] Wang Z., Hu Y., Meng Z., Luo H., Ni M. (2008). Novel mechanical antialiasing fiber-optic hydrophone with a fourth-order acoustic low-pass filter. Opt. Lett..

[27] Yuan W., Pang B., Bo J., Qian X. (2014). Fiber-optic sensor without polarization-induced signal fading. Microw. Opt. Technol. Lett..

[28] De Freitas J.M. (2011). Recent developments in seismic seabed oil reservoir nonitoring applications using fiber-optic sensing networks. Meas. Sci. Technol..

